# Spin generation via bulk spin current in three-dimensional topological insulators

**DOI:** 10.1038/ncomms10878

**Published:** 2016-03-02

**Authors:** Xingyue Peng, Yiming Yang, Rajiv R.P. Singh, Sergey Y. Savrasov, Dong Yu

**Affiliations:** 1 Department of Physics, University of California, One Shields Avenue, California 95616, USA

## Abstract

To date, spin generation in three-dimensional topological insulators is primarily modelled as a single-surface phenomenon, attributed to the momentum-spin locking on each individual surface. In this article, we propose a mechanism of spin generation where the role of the insulating yet topologically non-trivial bulk becomes explicit: an external electric field creates a transverse pure spin current through the bulk of a three-dimensional topological insulator, which transports spins between the top and bottom surfaces. Under sufficiently high surface disorder, the spin relaxation time can be extended via the Dyakonov–Perel mechanism. Consequently, both the spin generation efficiency and surface conductivity are largely enhanced. Numerical simulation confirms that this spin generation mechanism originates from the unique topological connection of the top and bottom surfaces and is absent in other two-dimensional systems such as graphene, even though they possess a similar Dirac cone-type dispersion.

## Abstract (web summary)

Future spintronic devices may exploit topological insulators, bulk-insulating materials possessing conductive surface states with orthogonally-locked electronic spin and momentum. Here, the authors propose a mechanism by which bulk spin currents drive surface spin accumulation in such a material.

## Graphical abstract

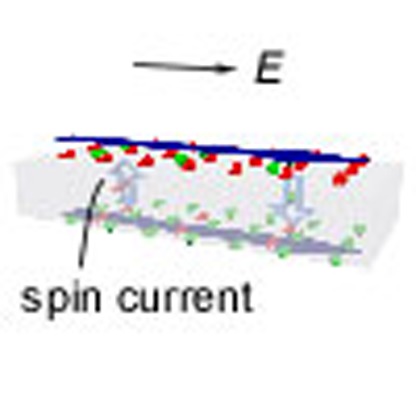

Topological insulators (TIs) have attracted world-wide attention because of their intriguing fundamental physics and exciting application opportunities in spintronics^1^. Three-dimensional (3D) TIs^2^,^3^ are of particular technological importance as the unique spin generation can be realized in single crystals rather than in complex heterogeneous structures^4^. TIs are considered as efficient spin generators^5^, yet the spin generation is generally regarded as a pure surface phenomenon. Namely, the electronic momentum and spin are locked at the TI surface, and a net charge current leads to a net spin polarization at the surface, whose magnitude is directly proportional to the charge current^6^. In this view, all physics occur independently at the top and bottom surfaces of a TI and the role of the bulk is passive, which simply separates the top and bottom surfaces. The surface conductivity is understood through density of states and scattering rate, just like in other two-dimensional (2D) systems such as graphene and 2D electron gas. The conductivity behaviour governs the spin generation on the surface of a 3D TI, and spin accumulation is merely a side product of conductivity. Although this interpretation of spin generation in TIs is most mathematically straightforward, it is far from satisfactory in the sense that the most amazing feature of a TI—surface-bulk correspondence does not explicitly enter this physical picture.

On the other hand, there is an alternative viewpoint of spin generation. The external electric field induces a transverse pure spin current through the bulk, which acts as a bridge for transporting spins between top and bottom surfaces. Opposite spins are thus accumulated on the two surfaces, which lead to charge current in the same direction of the electric field because of the opposite chirality of the momentum-spin textures on the top and bottom surfaces (Fig. 1a). An empirical formula for the bulk spin current can be written down as


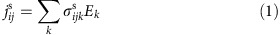


where *j*^s^ is the spin current density, *E* is the electric field and *σ*^s^ is the spin Hall conductivity tensor^7^. A system that is electrically insulating but can carry a pure spin current is termed a spin Hall insulator^8^. The bulk of a 3D TI has been demonstrated to be a spin Hall insulator because of its *Z*_2_ topological order^9^.

Analogous to Hall effect, the transverse spin Hall current leads to surface spin accumulation in a slab geometry. Yet unlike electric charge, spin is usually a nonconserved quantity in a spin Hall insulator. The ultimate spin accumulation induced on the surface closely depends on the spin relaxation mechanism. In the low disorder limit 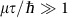 with *μ* being the Fermi level, 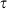 being the momentum relaxation time, it has been demonstrated that the spin relaxation time 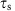 on the surface of a 3D TI is identical to the momentum relaxation time 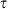 because of the momentum-spin locking, and the traditional Dyakonov–Perel spin relaxation is absent^10^. Charge-spin dynamics in the high disorder limit 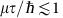, however, has rarely been discussed in the literature so far.

The exact behaviour of these spin transport coefficients under high disorder is of crucial importance to the application of 3D TI-based spintronic devices, because unlike the bulk, surface is extremely vulnerable to various kinds of defects, especially when placed in ambient environment. Even for a material that is generally considered ‘inert', the top most layer of atoms could still suffer from high concentration of impurities^11^,^12^. A clear physical model of charge and spin transport in this case is highly desired for the design of novel 3D TI-based spintronic devices.

In this article, we demonstrate that the unique topological connection of the surface bands in a 3D TI demands a pure spin current through the insulating bulk. Spins are thus generated and accumulated on the two surfaces. Sufficiently high surface disorder can suppress spin relaxation and result in an increase of the spin relaxation time 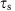 in a manner similar to the traditional Dyakonov–Perel mechanism. Consequently, both electro-spin susceptibility *κ*_*yx*_ (surface spin density *s*_*y*_ divided by the electric field *E*_*x*_) and the electrical conductivity *σ*_*xx*_ should increase with the increase of disorder, as illustrated in Fig. 1b.

## Results

### Spectral function and electrical conductivity

To begin with, we consider a realistic four-band tight-binding model^13^ built on a slab of a tetragonal lattice, as shown in Fig. 2a. The slab is infinite in *xy* directions and has a total number of *N*=10 layers in the *z* direction (*c*-axis). With four states on each site, the bulk Hamiltonian in the 3D **k**-space is


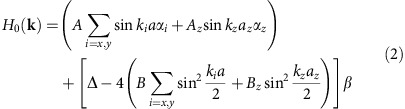


where *α*_*i*_(*i*=*x*, *y*, *z*), *β* are the Dirac matrices, *a* and *a*_*z*_ are the lattice constants in the *xy* and *z* directions, Δ is the mass term and *A*, *A*_*z*_, *B*, *B*_*z*_ are nearest-neighbour hopping amplitudes. In the slab configuration, inverse Fourier transform is performed in *z*-direction to comply with the finite thickness.

We use a typical 3D TI Bi_2_Se_3_ as our prototype and adopt parameters as obtained in ref. 2 to best fit the band structure of Bi_2_Se_3_. The resultant band structure of the surface is shown in Fig. 2b, which clearly has a Dirac cone near the Γ point. Owing to the *z*-inversion symmetry of the slab, all bands are doubly degenerate.

To account for surface disorder, atoms in the top and bottom layers of the slab are subject to a typical kind of impurity—vacancies. Each site at the surface has a probability of *c* to be occupied by a vacancy where the on-site energy is brought to infinity so as to forbid electrons from this site. As *c* may not be small, the first Born approximation does not apply. Here we adopted the coherent potential approximation (CPA) method for binary alloys^14^,^15^ in computing the Green's function *G*(**k**, *ω*) and self-energy Σ(*ω*). A typical spectral function −(1/*π*)Im*G*(**k**, *ω*) obtained by CPA at impurity concentration *c*=0.001 is plotted in Fig. 2c. The evolution of the spectral function with increasing impurity concentration is consistent with results obtained in ref. 13. Subsequently, transport coefficients were calculated via the standard linear response theory. More details of the numerical simulation can be found in the Methods section.

Figure 2c,d shows the electrical conductivity calculated from the CPA Green's function via the Kubo–Greenwood formalism. With an impurity concentration *c* ranging from 5 × 10^−4^ to 0.5, the Fermi level dependence of conductivity gets weaker and the magnitude of conductivity reaches a minimum at around *c*=0.006. Further increasing the impurity concentration leads to an increase of conductivity at a given Fermi level position. Such anomalous increase of conductivity with impurity concentration is difficult to understand based on a single surface model^16^,^17^, which suggests the essential role of the bulk of a 3D TI in surface conduction. In the following, we reveal that the anomalous increase of conductivity is a signature of a different type of spin dynamics and manifests a spin generation mechanism in 3D TIs.

### Spin generation via bulk spin current

To start discussions on spin dynamics, we notice that spin is not a predefined quantity in Hamiltonian (equation 2). Although common 3D TIs such as Bi_2_Se_3_ are known to have chiral spin texture on the surface states, the spin polarization is not 100% (ref. 18). Nevertheless, one can always talk about a pseudo-spin, which is defined to exactly match the energy eigenstates and has all essential features of the real spin^19^. Here we take the definition


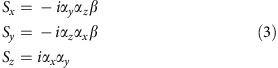


It can be verified that such definition satisfies all symmetry requirements of the real spin and shows a chiral spin texture near the Γ point, as shown in Fig. 3a. Note that a unique spin polarization can be specified for all points in the **k**-space except for those time-reversal invariant momenta (TRIM), where the Kramers theorem asserts the degeneracy of the two opposite spin polarizations.

With the above definition of spins, the bulk spin current density 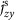 denoting the transport of *y*-spins in the *z*-direction can be defined as


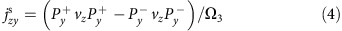


where 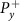 and 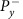 are spin projection operators in the +*y* and −*y* directions, *v*_*z*_ is the velocity operator in the *z* direction and Ω_3_ is the volume of the slab serving as a normalization factor. With the above expression, the spin Hall conductivity 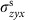 defined by


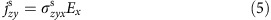


can be calculated via the standard linear response theory. Owing to the even symmetry of spin current *j*^s^ under time reversal 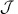, the resultant expression for the spin Hall conductivity is different from that for the electrical conductivity (Kubo–Greenwood formula), but contains a term which involves all states below the Fermi level. This term has been thoroughly reviewed in ref. 20 for the calculation of electrical conductivity in a 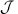-symmetry broken system and also discussed in a recently published article^21^ for the calculation of spin Hall conductivity. The emergence of this term in our system indicates the non-dissipative nature of the spin current, which has already been demonstrated possible for a wide class of traditional semiconductors^22^,^23^. We leave the details of derivation to the Methods section and plot the calculated spin Hall conductivity 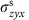 in Fig. 3b. It is clear that the magnitude of the spin Hall conductivity is independent of both the Fermi level position (must be within the bulk bandgap) and the surface impurity concentration.

Although the existence of a bulk spin current in 3D TI has been predicted analytically through topological argument^9^, a visualization of the spin transfer mechanism is not yet available so far. Neither has its relevance to the transport behaviour of the gapless surface states been studied ever. In the following, we present an intuitive picture of the spin transfer in a 3D TI slab and uncover its close relationship with the surface-bulk correspondence of a 3D TI.

We notice that despite the chiral spin texture over the entire Brillouin zone (BZ), only states with small magnitude of momentum are truly localized on the surface. Figure 3c,d shows the evolution of electronic wave functions as the wave vector **k** approaches the BZ boundary from the Γ point. It is seen that, beyond a certain point, electronic wave functions become extended through the entire bulk and the surface band has essentially merged into bulk bands. States beyond this merging point should be classified as bulk states although they lie on the same branch of energy sub-band as true surface states.

**Figure 1:**
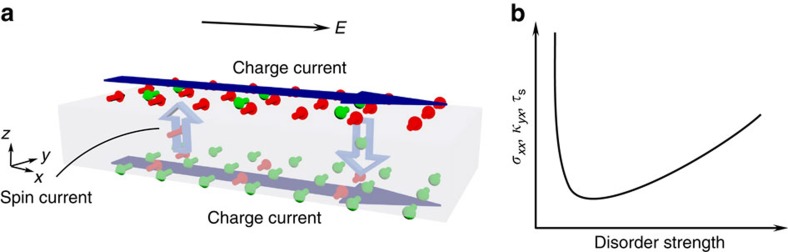
Proposed spin dynamics in a 3D TI. (**a**) An electric field induces a transverse pure spin current in the bulk. Consequently, opposite spins accumulate on the top and bottom surfaces, leading to a charge current according to the chiral momentum-spin texture. The small cylindrical arrows denote spins. The hollow vertical arrows indicate spin current. The long horizontal blue arrows indicate charge current. (**b**) The anomalous behaviour of transport coefficients proposed in this article. At a sufficiently high disorder level, conductivity *σ*_*xx*_, electro-spin susceptibility *κ*_*yx*_ and spin relaxation time *τ*_s_ should all have positive dependence on the disorder, in contrast with the well-known negative dependence in the low disorder limit.

**Figure 2:**
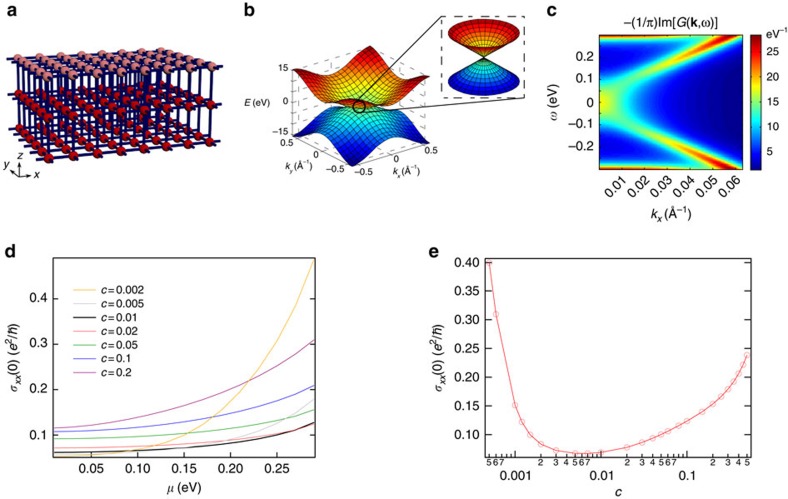
Model configurations and conductivity simulation. (**a**) The slab of tetragonal lattice is infinite in the *xy* directions and has a total number of *N*=10 layers in the *z* direction (*c*-axis of the lattice). Only the top three layers are shown here. There are four states on each site and only nearest-neighbour hopping is considered. The top and bottom layers are subject to vacancies, which are coloured pink in this figure. Each atom on these layers has a probability of *c* to be occupied by a vacancy and (1−*c*) to be intact. The on-site energy of a vacancy is brought to infinity to forbid electrons from entering this site. (**b**) The energy dispersion of the surface branch of a clean system. A Dirac cone exists around the Γ point. Parameters used for simulation: *A*=1 eV, *A*_*z*_=0.5 eV, *B*=2 eV, *B*_*z*_=0.4 eV, Δ=0.3 eV, *a*=5 Å. (**c**) The spectral function −(1/*π*)Im*G*(**k**, *ω*) obtained via CPA plotted along the Γ−X line at impurity concentration *c*=0.001. At higher concentrations, the **k**-dispersion first fades away and then slowly recovers, as discussed in ref. 13. (**d**) The DC conductivity of a single surface (*σ*_*xx*_(0)) plotted against the Fermi level position (*μ*). The impurity concentration *c* varies from 0.002 to 0.2. (**e**) Conductivity (*σ*_*xx*_(0)) plotted against the impurity concentration (*c*). The Fermi level position was fixed at *μ*=0.13 eV.

**Figure 3:**
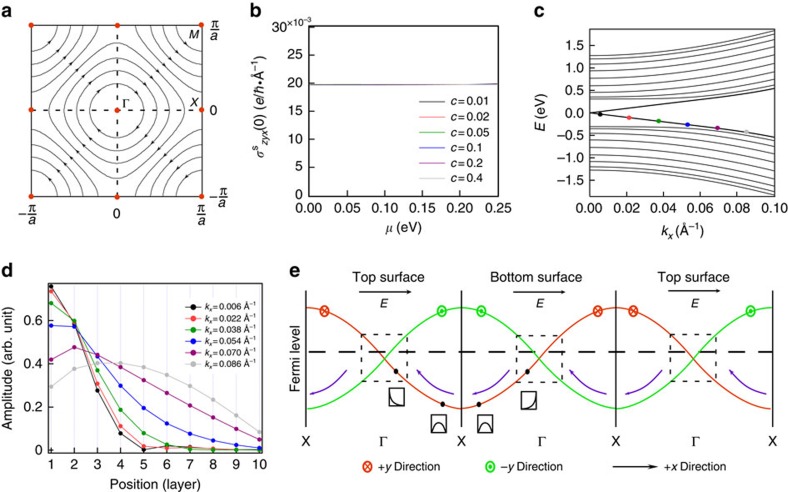
The spin generation mechanism. (**a**) The chiral spin texture over the entire BZ for the conduction band of the top surface. The spin orientations on TRIM points (red dots) are degenerate due to the Kramers theorem. (**b**) The DC spin Hall conductivity 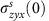 obtained from numerical simulation plotted against the Fermi level position *μ* at different impurity concentrations *c*. It is seen that the bulk spin current is independent of both Fermi level position and impurity concentration. (**c**) Energy dispersion for the top surface near the Γ point along Γ−X direction. The spin polarization of the coloured dots is in the + *y* direction. (**d**) The evolution of electronic wave functions along the coloured dots in **c**. As the magnitude of wave vector becomes larger, the wave function of an electron gradually evolves from being localized near the surface to extensive in the bulk. (**e**) A schematic plot for the spin generation mechanism in the extended BZ view. The drift motion along *x* direction in *k* space gives rise to spin transfer in the *z* direction of the real space, which results in a pure spin current through the bulk. The red/green arrows pointing into/out of the page indicate spin polarization. The purple arrows indicate the direction of drift motion of electrons under an electric field in the +*x* direction. The dashed horizontal line indicates the Fermi level position. The schematic drawing under the black dots denote the electronic wave functions. The dashed boxes denote the true surface state regions. It is essential that the Fermi level lies within the box regions for the spin transfer mechanism to apply.

Imagine applying a weak electric field to this system in the +*x* direction and examine the Γ−X line in the extended BZ view. Because of the inversion symmetry in the *z* direction, all bands are doubly degenerate. We notice, however, in order for the spin texture to be continuous, every top surface branch must be connected to the adjacent bottom surface branch and vice versa, as shown in Fig. 3e. This alternating structure exists across all TRIM points in our system, and is distinctively different from a normal band, which smoothly connects to itself at the BZ boundary. Consider an electron on the bottom surface with its spin polarized in +*y* direction. Under the driving of the electric field, this electronic state drifts to −*x* direction in *k* space and merges into the bulk valance band. Upon further drifting, this electron finally enters the top surface with its spin in +*y* direction unchanged. Simultaneously, an electron with spin polarized in the −*y* direction will drift from the top surface to bottom surface. The drift motion across the X point is similar to the Klein tunnelling of Dirac Fermions in the sense that, in order for a certain spin to be continuous, the electron must tunnel to another band rather than return to its original band. Overall, each of these processes corresponds to a unit spin-pair exchange between the bottom surface and the top surface. Thus, a longitudinal electric field induces a transverse pure spin current through the bulk, which plays the role of a spin injector for the two surfaces, as described in Fig. 1a. During this process, it is essential that the Fermi level lies within the gap, because there exists another pair of merging points near the conduction band edge. If the Fermi level is above these points as well, there would be an opposite process that leads to the cancelation of net spin current. This is of course consistent because the system in this case is not a TI any more.

### Spin relaxation on the surface

**Figure 4:**
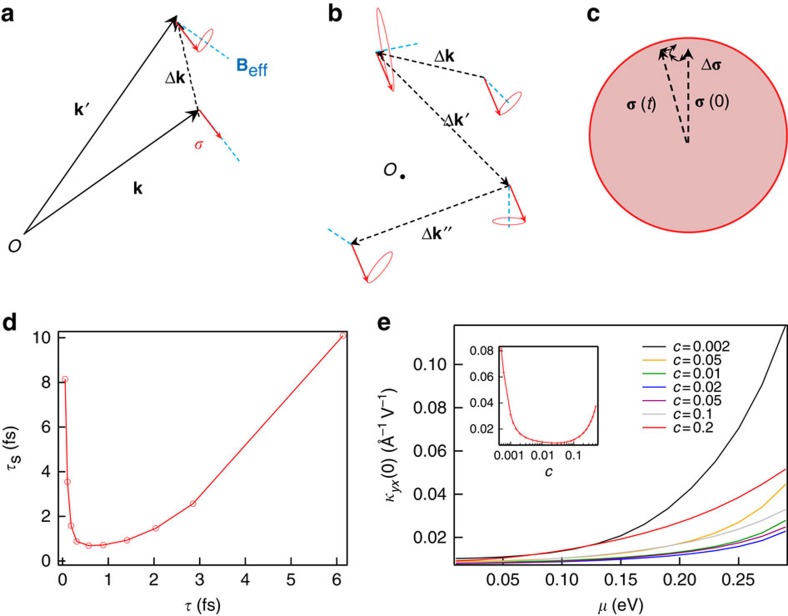
Spin relaxation mechanism under high level of nonmagnetic disorder. (**a**) When scatterings are frequent, an electron suffers from rapid momentum change where the electronic spin does not have time to follow the instantaneous energy eigenstate given by adiabatic perturbation theory. Instead, the electronic spin precesses about the instantaneous energy eigenstate, which serves as an effective magnetic field **B**_eff_. (**b**) Frequent scatterings constantly change the precession axis of spin. During the interval of two consecutive scatterings the spin can only precess for a small angle. (**c**) The spin ends up doing a random walk on a unit sphere. The more frequent the scatterings are, the less efficient this random walk is, and consequently spin can preserve its original direction for a longer time. (**d**) The numerical simulation result for spin relaxation time 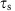 and momentum relaxation time 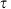 at Fermi level *μ*=0.13 eV. As expected, when disorder is high, 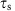 and 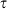 have an inverse dependence as in the traditional Dyakonov–Perel spin relaxation mechanism. (**e**) The simulated DC electro-spin susceptibility *κ*_*yx*_(0) plotted against the Fermi level *μ* at different impurity concentrations. The inset is *κ*_*yx*_(0) at a fixed Fermi level *μ*=0.13 eV versus impurity concentration *c*. It is clear that under high disorder, the accumulated spin density increases with impurity concentration.

Unlike charge, spin is not a conserved quantity in our system. The spins injected onto the surface suffer from immediate relaxation. The scenario is slightly different from both the Hall effect and the 2D spin Hall effect. In 3D, the spin relaxation is actually necessary for the system to reach a steady state, as detailed in Supplementary Note 1. The ultimate spin density accumulated on the surface is determined by the spin relaxation time 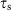. In the following, we provide an intuitive physical picture of the spin relaxation dynamics under the eigenbasis defined by *H*_0_. More details of this picture can be found in Supplementary Note 2. This is essentially an interaction picture that splits the Hamiltonian into a free part *H*_0_ and an interaction part *U*. Because of the momentum-spin locking, each electron senses an effective magnetic field **B**_eff_ according to its wave vector **k**. When an electron with its spin aligned with **B**_eff_ suffers from a momentum change *ħ*Δ**k** because of the scattering of an impurity potential, its spin may no longer align with the new **B**_eff_. If scatterings are rare, that is, the time it takes for momentum to change by a unit amount is long, the adiabatic perturbation theory predicts that the new spin must evolve to the new energy eigenstate, that is, rotate to the direction of the new **B**_eff_. If scatterings are frequent, however, the spin does not have time to follow **B**_eff_ and will precess about the instantaneous **B**_eff_, as shown in Fig. 4a. Frequent scatterings constantly change the precession axis and the spin ends up doing a random walk on a unit sphere, as shown in Fig. 4b,c. The more frequent momentum scattering is, the less effective the random walk is, and the spin will preserve its original direction for a longer time. Therefore, the spin relaxation time 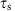 inversely depends on the momentum relaxation time *τ*, just like in the traditional Dyakonov–Perel mechanism^24^. One point to note is that as disorder is only present on the surface, states outside the dashed box of Fig. 3e are unaffected by scattering and the previously discussed spin transfer mechanism remains valid even under strong surface disorder.

If the above physical picture is correct, the ultimate spin density accumulated on the surface should increase with the increase of disorder. We calculated the spin relaxation time 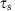 and electro-spin susceptibility *κ*_*yx*_ via standard linear response theory. The results are shown in Fig. 4d,e, which perfectly agree with the expectation. Combined with the fact that velocity operator is proportional to spin on the surface, it is not difficult to understand the anomalous increase of conductivity as well.

## Discussion

**Figure 5:**
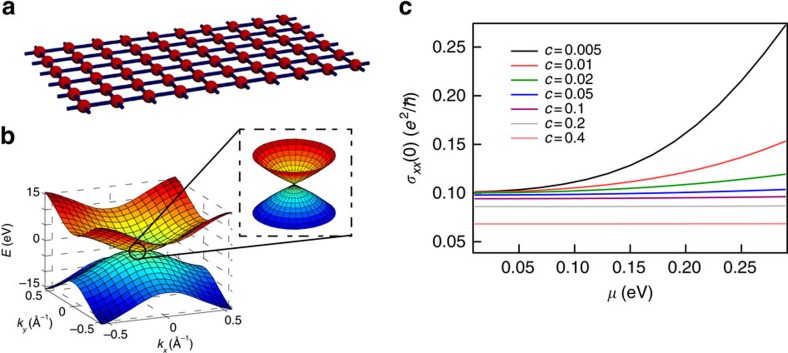
Simulation for a single layer with the same model to illustrate the importance of spin dynamics in our previous system. (**a**) The geometry of the system. Each atom on this layer has a probability of *c* to be occupied by a vacancy and (1−*c*) to be intact. The on-site energy of a vacancy is brought to infinity. Only nearest-neighbour hopping is allowed. (**b**) The energy dispersion of this single layer system. Simulation parameters are identical to before except for the mass term Δ is set to zero in order to have a Dirac cone near the Γ point. The band structure looks almost identical to the surface bands of the 3D TI slab discussed before. (**c**) The DC conductivity *σ*_*xx*_(0) of this single layer versus Fermi level at different impurity concentrations. Despite the similarity in band structures, this single layer does not have anomalous increase of conductivity at high disorder, in contrast with our previous system.

It is worth noting that, in contrast to a common belief, the behaviour of conductivity of a Dirac system under high disorder is not simply governed by the dispersion relationship. The anomalous increase of conductivity is closely related to the spin generation and relaxation mechanism. To illustrate this point, we calculated the conductivity of a single layer of atoms within the same model (Fig. 5a). By setting the mass term Δ=0, the band structure of this 2D system has almost identical shape as the previous surface state, as shown in Fig. 5b, but the spin generation mechanism discussed above is obviously absent. With the increase of the impurity concentration, the conductivity of such system monotonically decreases towards zero, even in the high disorder range. Similar behaviour has also been shown in graphene^25^,^26^, where no anomalous increase of conductivity was found.

The spin dynamics in a 3D TI under strong surface disorder makes delicate connection with the scenario of a 2D TI. In fact, it is straightforward to apply the previously discussed spin transfer mechanism to a 2D TI, and obtain the universal quantized spin Hall conductivity 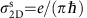. Different from the 3D case, in the edge channel of a 2D TI, the spin (understood as pseudo-spin when necessary) is a conserved quantity, which does not relax. Consequently, the electro-spin susceptibility is infinite, which means no external field is needed to support the edge spin accumulation and charge current. The quantized and finite channel conductivity *e*^2^/*h* is actually a contact effect, while the channel itself is dissipationless^27^. On the surface of a 3D TI, however, spin is not conserved due to an additional angular degree of freedom of the wave vector **k**. The electro-spin susceptibility is thus finite and transport is dissipative. Strong surface disorder greatly suppresses spin relaxation and brings the system closer to the situation of a 2D TI, leading to a more efficient spin generation. In this view, strong surface disorder can be beneficial for spintronic devices, in contrast with the common belief. Technically, it is obviously of more convenience to induce high disorder on a surface than to make it pure and pristine.

Experimentally, the anomalous increase of conductivity in 3D TIs has already been hinted by results from several groups, yet researchers do not generally regard it as an intrinsic property of the TI surface. Field effect measurements in TIs have often shown a high minimum conductivity even when the Fermi level is tuned to the charge neutral point^28^,^29^,^30^,^31^,^32^, which is much larger than expected by normal transport theory assuming low disorder^16^,^17^. Although they are often attributed to bulk conduction^29^ or surface electron puddle formation^30^,^33^, a closer look at these models reveals several problems, which are discussed in details in Supplementary Note 3. Theory presented in this paper, however, provides a simple and natural way to understand these observations. Recently, it has been directly observed in exfoliated BiSbTeSe_2_ nanoflakes that after argon ion milling treatment to create more surface defects, the sample becomes more conductive^34^, although this effect was not understood.

Our simulation results also suggest that under strong surface disorder, with the magnitude of transport coefficients increased, their Fermi level sensitivity has dropped, which is also beneficial for the design of 3D TI-based spintronic devices. This is because in ambient environment, the Fermi level on the surface is subject to unintentional change because of contamination and degradation^35^,^36^. Sensitive Fermi level dependence renders the device less stable and robust in air.

## Methods

### CPA and self-energy

The numerical calculation results were obtained via CPA and the standard linear response theory. In the following, we use convention *ħ*=1. The Hamiltonian of a clean lattice in the main text can be written in a block diagonal form


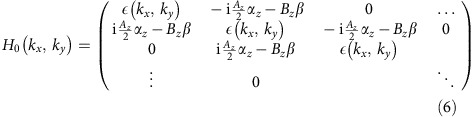


where each element in this matrix is a 4 × 4 matrix. The *N* rows and columns denote the *N* layers of the slab. The on-site energy 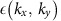 takes the form


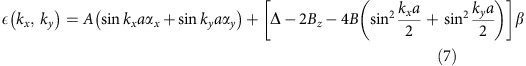


Overall, the Hamiltonian is 4*N* × 4*N*. Diagonalizing this matrix gives the band structure of a clean lattice without impurities.

We consider impurities of an on-site scalar potential *U* on the top and bottom layers, which takes the form of


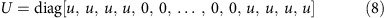


Each site has a probability of *c* subject to potential *U* and probability (1−*c*) subject to potential 0, which makes the entire system essentially a binary alloy. Such configuration assumes non-physical correlation between the appearance of an impurity on the top and bottom layers. Yet from practical consideration, for a sufficiently thick slab, the crosstalk between the top and bottom layers should vanish, which justifies the binary alloy model of the above.

In CPA, the configurationally averaged impurity potential is denoted by a **k**-independent self-energy Σ(*ω*). In the binary alloy case, Σ(*ω*) is determined by the iterative equation


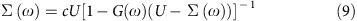


where *G*(*ω*) is the on-site Green's function


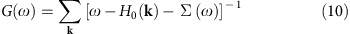


Because of the symmetry of this problem, Σ(*ω*) is actually a scalar on the top and bottom layers only. The real and imaginary parts of the self-energy are plotted in Supplementary Fig. 1.

### Conductivity

The velocity operator **v** takes the form


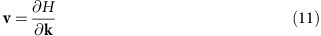



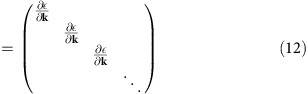


According to the Kubo formula, the direct current (DC) conductivity at *T*=0 is


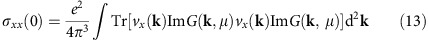


The conductivity of the top surface is half of this value


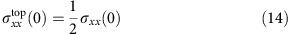


### Definition of spin operators and the chiral spin texture

Spin is not a predefined quantity in the original Hamiltonian. Unless we are interested in a direct coupling to an external magnetic field, the four state basis vectors can be thought to have arbitrary spin polarizations, which is essentially a ‘pseudo-spin'. Even in real TIs such as Bi_2_Se_3_, the pseudo-spin does not exactly match the real spin. Nevertheless, the definition of a ‘pseudo-spin' **S** must satisfy a couple of restrictions: (i) **S** is an Hermitian operator, 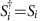. (ii) The components of **S** satisfy the anti-commutation rules, 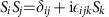. (iii) **S** is a pseudo-vector. It transforms like a vector under in-plane (*xy*) rotation but does not flip sign under space inversion, *βS*_*i*_*β*=*S*_*i*_. (4) To comply with the chiral surface spin texture, we require **S** be polarized along *y*-direction when *k*_*y*_=0. Thus, [*S*_*y*_, *H*(*k*_*x*_, 0)]=0.

Note that we do not include *ħ*/2 in our definition, so this pseudo-spin has dimension 1 instead of angular momentum. It can be easily verified that the following expressions are a good representation of ‘spin' in our system.


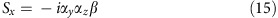



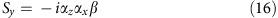



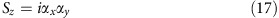


The spin in an arbitrary direction *θ* within the *xy* plane is thus


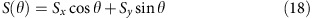


If there exists an angle *θ* for an arbitrary point in *k*-space (*k*_*x*_, *k*_*y*_) such that


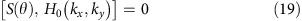


then *S*(*θ*) and *H*_0_(*k*_*x*_, *k*_*y*_) have common eigenstates and the energy eigenstates can be assigned a unique spin polarization. It is not difficult to obtain


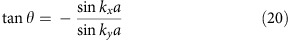


When the magnitude of **k** is small, we recover to the well-known chiral spin texture


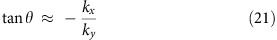


whereas when **k** gets large, the spin texture deforms to adapt to the tetragonal symmetry of the BZ, as shown in the main text.

### Surface spin density and electro-spin susceptibility

The spin density accumulated on the top surface is


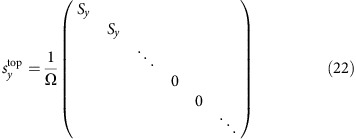


At *T*=0 the electro-spin susceptibility is calculated similar to the electrical conductivity


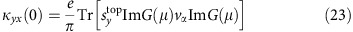



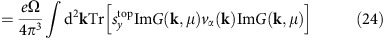


Note that the definition of 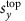 contains a factor of 1/Ω and thus the above expression is actually independent of the box size Ω.

### Bulk spin current and spin Hall conductivity

The z-position operator is


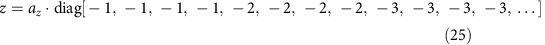


Thus, the z-velocity operator


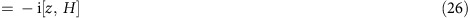



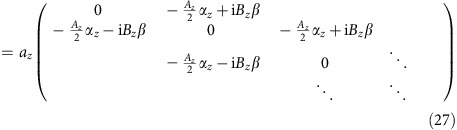


As we are interested in the flux across a certain intermediate layer, we restrict the velocity operator to be only between two adjacent layers in the middle


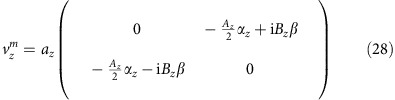


To discuss the spin current, we need to define a spin projection operator


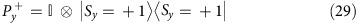



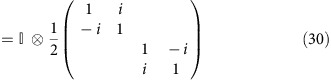



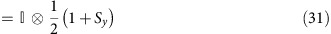



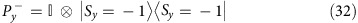



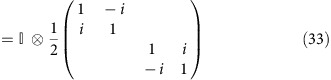



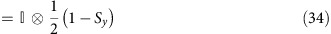


The spin current operator is then


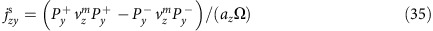



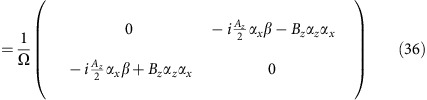


Under time reversal


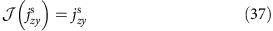


which is different from charge current or spin density. Thus, the spin Hall conductivity is not only determined by the Green's function at the Fermi level but also contains an additional term contributed by all states occupied^20^,^21^.


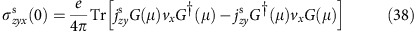



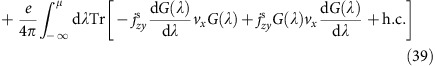



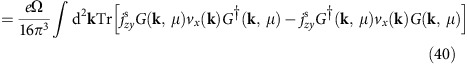



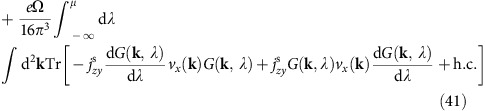


Again because of the 1/Ω factor in the definition of 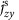, the above expression is independent of the box size Ω.

### Spin relaxation time

Imagine applying a pulse electric field to our system


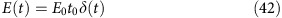


Empirically, the induced spin has the asymptotic form


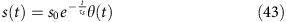


where the function *θ*(*t*)=0 when *t*<0 and *θ*(*t*)→1 as *t*→+∞. Consider the Fourier transform


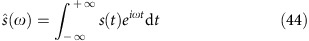



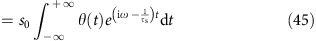


For sufficiently small *ω*, the details of the rising part of *s*(*t*) characterized by *θ*(*t*) becomes unimportant, thus we replace *θ*(*t*) with the step function Θ(*t*) and obtain


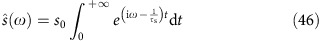



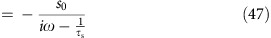


The Fourier transform of the electric field is just a constant


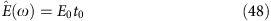


Thus, the electro-spin susceptibility


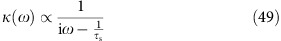


and the spin relaxation time can be extracted as


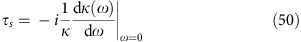



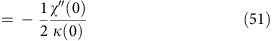


It is necessary to point out that the spin relaxation process can be thought as an eigen mode with a complex frequency ω* on the lower half plane. ω* is a pole of the response function *χ*(ω*)=∞. The spin relaxation time is determined by the imaginary part of the pole closest to the real axis


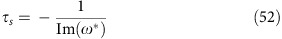


The equation 51 is based on the low-frequency expansion of *χ*(*ω*), which may not give the exact pole position, and ref. 10 has shown that for a perfect Dirac-cone dispersion, the spin relaxation time found by the exact pole is twice as the value found by low frequency expansion. Nevertheless, apart from an order 1 factor, low-frequency expansion should give a reasonable estimate of the true spin relaxation time.

## Additional information

**How to cite this article:** Peng, X. *et al*. Spin generation via bulk spin current in three-dimensional topological insulators. *Nat. Commun.* 7:10878 doi: 10.1038/ncomms10878 (2016).

## Supplementary Material

Supplementary Information Supplementary Figures 1, Supplementary Notes 1-3 and Supplementary References

## References

[b1] Hasan M. Z. & Kane C. L. Colloquium: topological insulators. Rev. Mod. Phys. 82, 3045–3067 (2010).

[b2] Zhang H. J. . Topological insulators in Bi_2_Se_3_, Bi_2_Te_3_ and Sb_2_Te_3_ with a single Dirac cone on the surface. Nat. Phys. 5, 438–442 (2009).

[b3] Chen Y. L. . Experimental realization of a three-dimensional topological insulator, Bi_2_Te_3_. Science 325, 178–181 (2009). 19520912 10.1126/science.1173034

[b4] Konig M. . Quantum spin Hall insulator state in HgTe quantum wells. Science 318, 766–770 (2007). 17885096 10.1126/science.1148047

[b5] Pesin D. & MacDonald A. H. Spintronics and pseudospintronics in graphene and topological insulators. Nat. Mater. 11, 409–416 (2012). 22522641 10.1038/nmat3305

[b6] Li C. H. . Electrical detection of charge-current-induced spin polarization due to spin-momentum locking in Bi_2_Se_3_. Nat. Nanotech 9, 218–224 (2014). 10.1038/nnano.2014.16 24561354

[b7] Hasan M. Z. & Moore J. E. Three-dimensional topological insulators. Annu. Rev. Cond. Mat. Phys. 2, 55–78 (2011).

[b8] Murakami S., Nagaosa N. & Zhang S. C. Spin-Hall insulator. Phys. Rev. Lett. 93, 156804 (2004). 15524922 10.1103/PhysRevLett.93.156804

[b9] Kane C. L. & Mele E. J. Z(2) topological order and the quantum spin Hall effect. Phys. Rev. Lett. 95, 146802 (2005). 16241681 10.1103/PhysRevLett.95.146802

[b10] Xin L. & Sinova J. Reading charge transport from the spin dynamics on the surface of a topological insulator. Phys. Rev. Lett. 111, 166801 (2013). 24182290 10.1103/PhysRevLett.111.166801

[b11] Yashina L. V. . Negligible surface reactivity of topological insulators Bi_2_Se_3_ and Bi_2_Te_3_ towards oxygen and water. ACS Nano 7, 5181–5191 (2013). 23679000 10.1021/nn400908b

[b12] Kong D. S. . Rapid surface oxidation as a source of surface degradation factor for Bi_2_Se_3_. ACS Nano 5, 4698–4703 (2011). 21568290 10.1021/nn200556h

[b13] Schubert G., Fehske H., Fritz L. & Vojta M. Fate of topological-insulator surface states under strong disorder. Phys. Rev. B 85, 201105 (2012).

[b14] Velicky B. Theory of electronic transport in disordered binary alloys - coherent-potential approximation. Phys. Rev. 184, 614–627 (1969).

[b15] Yonezawa F. & Morigaki K. Coherent potential approximation: basic concepts and applications. Prog. Theo. Phys. Supp. 53, 1–76 (1973).

[b16] Culcer D., Hwang E. H., Stanescu T. D. & Das Sarma S. Two-dimensional surface charge transport in topological insulators. Phys. Rev. B 82, 155457 (2010).

[b17] Li Q. Z., Rossi E. & Das Sarma S. Two-dimensional electronic transport on the surface of three-dimensional topological insulators. Phys. Rev. B 86, 235443 (2012).

[b18] Yazyev O. V., Moore J. E. & Louie S. G. Spin polarization and transport of surface states in the topological insulators Bi_2_Se_3_ and Bi_2_Te_3_ from first principles. Phys. Rev. Lett. 105, 266806 (2010). 21231702 10.1103/PhysRevLett.105.266806

[b19] Shen S.-Q. Topological Insulators: Dirac Equation in Condensed Matters Ch. 2, Solid-State Sciences, Springer (2012).

[b20] Nagaosa N., Sinova J., Onoda S., MacDonald A. H. & Ong N. P. Anomalous Hall effect. Rev. Mod. Phys. 82, 1539–1592 (2010).

[b21] Sinova J., Valenzuela S. O., Wunderlich J., Back C. H. & Jungwirth T. Spin Hall effects. Rev. Mod. Phys. 87, 1213–1259 (2015).

[b22] Murakami S., Nagaosa N. & Zhang S. C. Dissipationless quantum spin current at room temperature. Science 301, 1348–1351 (2003). 12907808 10.1126/science.1087128

[b23] Murakami S., Nagaosa N. & Zhang S. C. SU(2) non-Abelian holonomy and dissipationless spin current in semiconductors. Phys. Rev. B 69, 235206 (2004).

[b24] Dyakonov M. I. & Perel V. I. Spin relaxation of conduction electrons in noncentrosymmetric semiconductors. Sov. Phys. Solid State 13, 3023–3026 (1972).

[b25] Peres N. M. R., Guinea F. & Castro Neto A. H. Electronic properties of disordered two-dimensional carbon. Phys. Rev. B 73, 125411 (2006).

[b26] Nilsson J., Castro Neto A. H., Guinea F. & Peres N. M. R. Electronic properties of bilayer and multilayer graphene. Phys. Rev. B 78, 045405 (2008).

[b27] Bernevig B. A., Hughes T. L. & Zhang S. C. Quantum spin Hall effect and topological phase transition in HgTe quantum wells. Science 314, 1757–1761 (2006). 17170299 10.1126/science.1133734

[b28] Checkelsky J. G., Hor Y. S., Cava R. J. & Ong N. P. Bulk band gap and surface state conduction observed in voltage-tuned crystals of the topological insulator Bi_2_Se_3_. Phys. Rev. Lett. 106, 196801 (2011). 21668185 10.1103/PhysRevLett.106.196801

[b29] Sacepe B. . Gate-tuned normal and superconducting transport at the surface of a topological insulator. Nat. Commun. 2, 575 (2011). 22146394 10.1038/ncomms1586 PMC3247814

[b30] Kim D. . Surface conduction of topological dirac electrons in bulk insulating Bi_2_Se_3_. Nat. Phys. 8, 459–463 (2012).

[b31] Steinberg H., Gardner D. R., Lee Y. S. & Jarillo-Herrero P. Surface state transport and ambipolar electric field effect in Bi_2_Se_3_ nanodevices. Nano Lett. 10, 5032–5036 (2010). 21038914 10.1021/nl1032183

[b32] Seung Sae H., Cha J. J., Desheng K. & Cui Y. Ultra-low carrier concentration and surface-dominant transport in antimony-doped Bi_2_Se_3_ topological insulator nanoribbons. Nat. Commun. 3, 757 (2012). 22453830 10.1038/ncomms1771

[b33] Adam S., Hwang E. H., Galitski V. M. & Das Sarma S. A self-consistent theory for graphene transport. Proc. Natl Acad. Sci. USA 104, 18392–18397 (2007). 18003926 10.1073/pnas.0704772104 PMC2141788

[b34] Banerjee K. . Defect-induced negative magnetoresistance and surface state robustness in the topological insulator BiSbTeSe_2_. Phys. Rev. B 90, 235427 (2014).

[b35] Park S. R. . Quasiparticle scattering and the protected nature of the topological states in a parent topological insulator Bi_2_Se_3_. Phys. Rev. B 81, 041405 (R) (2010).

[b36] Hsieh D. . Observation of time-reversal-protected single-dirac-cone topological-insulator states in Bi_2_Te_3_ and Sb_2_Te_3_. Phys. Rev. Lett. 103, 146401 (2009). 19905585 10.1103/PhysRevLett.103.146401

