# Supplementary material for: Spin generation via bulk spin current in three-dimensional topological insulators
Source: Nat Commun. 2016 Mar 2;7:10878. doi: 10.1038/ncomms10878 (PMC4778060; doi:10.1038/ncomms10878)
Supplement: Supplementary Information — Supplementary Figures 1, Supplementary Notes 1-3 and Supplementary References [file ncomms10878-s1.pdf]

Supplementary Figures

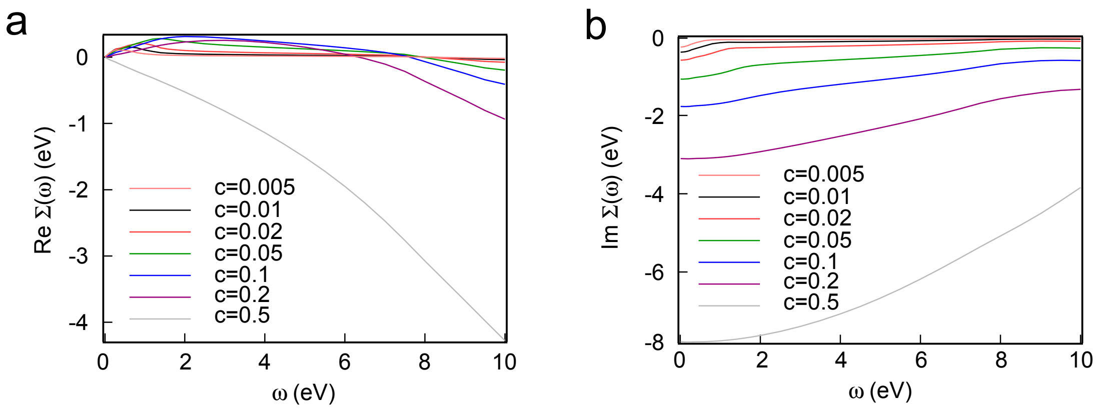

Supplementary Figure 1: The self-energy $\Sigma(\omega)$ computed for the 3D TI slab as discussed in the Methods section of the main text. **a,** The real part and **b,** The imaginary part.

Supplementary Note 1: Spin non-conservation and the role of spin relaxation in the spin Hall effect

As pointed out in the main text, spin is not a conserved quantity in this system. However, the non-conservation of spin does not completely break off the connection between spin density and spin current density. It only requires that a spin relaxation term be added to the continuity equation and taken with care. The bulk of our system is disorder-free where time reversal symmetry prohibits any spin accumulation at a steady state, thus spin accumulation and relaxation can only happen on the surface. An empirical equation for surface spin density s and bulk spin current j^s can be written down as

$$\frac{\mathrm{d}s}{\mathrm{d}t} = j^s - R(s) \tag{1}$$

where $R(s)$ is the surface spin relaxation rate which can be roughly expressed as s/τ_s. At a steady state, one has

$$j^s = R(s) \approx \frac{s}{\tau_s} \tag{2}$$

from which the accumulated surface spin density s is determined. If there were no such spin flip term, the system would never reach a steady state.

The situation for the spin Hall effect is subtly different from the Hall effect. In the Hall effect, the accumulated surface charge causes a lateral voltage drop to resist further accumulation of charge, such that at a steady state the lateral current $j \equiv 0$. In the spin Hall effect, however, the spin accumulation does not lead to any counter force for the spin current, and there is a persistent bulk spin current as long as the longitudinal electric field exists. This term has to be canceled by a spin flip term to reach the steady state.

The situation is also different from the 2D case. In the main text, we have argued that for the steady state of a 2D quantum spin Hall system there is actually no voltage drop along the conduction channel, but only across the contact. Therefore the lateral spin current $j^s \equiv 0$ similar to the Hall effect case. Thus it's possible to define a conserved spin current for a 2D system, but not for a 3D system where the voltage drops across the system itself.

Among the literature of spin Hall effect, some [1] did attempt to define a conserved spin component. Yet from the above argument it seems that in the spin Hall effect, a properly defined, physically observable "spin" which manifests itself as a surface spin accumulation should be nonconserved.

Supplementary Note 2: About the Dyakonov-Perel spin relaxation mechanism

A very tricky question regarding the Dyakonov-Perel spin relaxation mechanism in our system is the lack of $\mathbf{k}$ space. Under high disorder $\epsilon_F\tau \lesssim 1$, the impurity potential cannot be regarded as a perturbation thus wave vector $\mathbf{k}$ becomes an ill-defined quantity. Talking about the spin precessional random walk in this situation seems an unjustified story.

However, the lack of k-space is only true when we treat $H_0 + U$ as a whole. The spin random walk picture of D-P spin relaxation mechanism is actually an interaction picture which splits the Hamiltonian into a free part H_0 and an interaction part U. H_0 provides the energy eigenstate bases while U accounts for the time evolution of the wave function. The interaction Hamiltonian U does not have to be much smaller than H_0. Wave vector $\mathbf{k}$ is perfectly defined for H_0, which justifies the language of spin random walk.

To be more specific, we compare the situation of our system to a traditional 2DEG with Rashba spin splitting which is known to exhibit D-P mechanism. The Hamiltonian in this case is

$$H = H_b(\mathbf{k}) + H_s + U \tag{3}$$

where $H_b(\mathbf{k})$ is a spin-independent band energy, H_s is the spin splitting energy which can be expressed as $\hbar v_F\sigma\cdot\mathbf{k}$, U is the scattering term. Here v_F is just a parameter with no meaning of "Fermi velocity". The criterion for D-P mechanism is $|H_b| \gg |U| \gg |H_s|$ or $H_b \gg \hbar/\tau \gg \hbar v_F k$. The latter part of this criterion simply means the spin splitting structure is completely blurred by scattering. While on a 3D TI surface the entire band Hamiltonian is just the spin splitting energy, it certainly means a complete destruction of the k-space. The only difference from the traditional case is the lack of H_b term. However, we will argue in the following that H_b is not essential to the D-P mechanism.

We investigate the evolution of the system during a time t. We divide t into a lot of infinitesimal intervals

$$t = \Delta t_1 + \Delta t_2 + ... + \Delta t_N \tag{4}$$

The time evolution operator correspondingly breaks into

$$\mathrm{e}^{-\mathrm{i}\frac{H}{\hbar}t} = \mathrm{e}^{-\mathrm{i}\frac{H}{\hbar}\Delta t_1}\mathrm{e}^{-\mathrm{i}\frac{H}{\hbar}\Delta t_2}...\mathrm{e}^{-\mathrm{i}\frac{H}{\hbar}\Delta t_N} \tag{5}$$

Each interval can be separated in terms of the three terms of H

$$\mathrm{e}^{-\mathrm{i}\frac{H}{\hbar}\Delta t} = \mathrm{e}^{-\mathrm{i}\frac{H_b}{\hbar}\Delta t}\mathrm{e}^{-\mathrm{i}\frac{H_s}{\hbar}\Delta t}\mathrm{e}^{-\mathrm{i}\frac{U}{\hbar}\Delta t} \tag{6}$$

Now we consider an initial state

$$|\mathbf{k}\rangle \otimes |\sigma\rangle \tag{7}$$

with momentum $\mathbf{k}$ and spin in the σ direction ($|\sigma\rangle$'s are actually the coherent states of spin which form an over complete set in the spin space).

Acting $\mathrm{e}^{-\mathrm{i}\frac{U}{\hbar}\Delta t}$ on $|\mathbf{k}\rangle \otimes |\sigma\rangle$ will scatter it to a different $\mathbf{k}'$ with the amplitude determined by $U_{\mathbf{k}'\mathbf{k}}$

$$\mathrm{e}^{-\mathrm{i}\frac{U}{\hbar}\Delta t}|\mathbf{k}\rangle \otimes |\sigma\rangle = \sum_{\mathbf{k}'}\left(\delta_{\mathbf{k}'\mathbf{k}} - \mathrm{i}\frac{U_{\mathbf{k}'\mathbf{k}}}{\hbar}\Delta t\right)|\mathbf{k}'\rangle \otimes |\sigma\rangle \tag{8}$$

but leaving the spin vector σ unchanged.

Acting $\mathrm{e}^{-\mathrm{i}\frac{H_s}{\hbar}\Delta t}$ on $|\mathbf{k}\rangle \otimes |\sigma\rangle$ will precess the spin vector about the axis $\mathbf{k}$ by an angle

$$\mathrm{e}^{-\mathrm{i}\frac{H_s}{\hbar}\Delta t}|\mathbf{k}\rangle \otimes |\sigma\rangle = |\mathbf{k}\rangle \otimes |\sigma + 2v_F\mathbf{k}\Delta t \times \sigma\rangle \tag{9}$$

but leaving the momentum $\mathbf{k}$ unchanged.

Acting $\mathrm{e}^{-\mathrm{i}\frac{H_b}{\hbar}\Delta t}$ on $|\mathbf{k}\rangle \otimes |\sigma\rangle$ does not change anything but simply induces a phase factor

$$\mathrm{e}^{-\mathrm{i}\frac{H_b}{\hbar}\Delta t}|\mathbf{k}\rangle \otimes |\sigma\rangle = \mathrm{e}^{-\mathrm{i}\frac{H_b(\mathbf{k})}{\hbar}\Delta t}|\mathbf{k}\rangle \otimes |\sigma\rangle \tag{10}$$

Now we may assign each time interval Δt_i an available $\mathbf{k}_i$ to form an integral path

$$\mathbf{k}_1 \times \Delta t_1 \to \mathbf{k}_2 \times \Delta t_2 \to \mathbf{k}_3 \times \Delta t_3 \to ... \to \mathbf{k}_N \times \Delta t_N \tag{11}$$

The final state is just a sum over all paths.

Consider two extreme cases: (1) $|H_s| \gg |U|$. In this case the spin vector precesses by an appreciable angle far before the momentum has an appreciable probability to get scattered to a different value. The scattering is essentially an adiabatic rotation of the spin vector to the new energy eigenstate. Hence spin relaxation and momentum relaxation are bound together and we have $\tau_s = \tau$. (2) $|H_s| \ll |U|$. In this case the momentum gains an appreciable probability to be scattered to a different value far before the spin vector precesses by an appreciable angle. This will result in the precessional random walk picture of D-P mechanism. We thus expect $\tau_s \sim 1/\tau$.

The H_b term, however, will cause some restriction to the above picture through the additional phase factor $\mathrm{e}^{-\mathrm{i}\frac{H_b(\mathbf{k})}{\hbar}\Delta t}$. Consider a virtual variation to the path (11): we slightly change the lengths of Δt_i and Δt_{i+1} to $\Delta t_i + \delta$ and $\Delta t_{i+1} - \delta$. Since we have assumed $|H_b| \gg |H_s|$ and $|H_b| \gg |U|$, if $H_b(\mathbf{k}_i) \neq H_b(\mathbf{k}_{i+1})$, we can choose the value of δ such that the amplitude contributions by H_s and U remain almost unchanged but the phase factor by H_b changes drastically. Consequently, summing over these paths will result in cancelation. The only exception is paths with

$$H_b(\mathbf{k}_1) = H_b(\mathbf{k}_2) = ... = H_b(\mathbf{k}_N) \tag{12}$$

where the contribution of H_b becomes a trivial global phase factor. Therefore, we see that the presence of the H_b term simply restricts available paths to those on the constant energy contour of $H_b(\mathbf{k})$.

Now for the surface of a 3D TI without the H_b term, we simply remove the restriction that $\mathbf{k}$ must stay on a constant energy contour. The precessional random walk picture still holds even though there is no semi-classical orbital motion.

Supplementary Note 3: Difficulties in existing models for the minimum conductivity in 3D TIs

In this section we address in details why the two currently existing models do not explain the minimum conductivity satisfactorily.

In [2], the authors showed a resistance peak of 70 Ω while tuning the gate voltage applied to a 10 nm-thick Bi_2Se_3 thin film. Considering the 1 : 8 aspect ratio of the conduction channel, this resistance converts to a 560 Ω square resistivity ($\sim 50e^2/h$). The authors attributed this conductance to electrons hopping in a bulk impurity band. Charged impurities in Bi_2Se_3 are believed to have a relatively large Bohr radius $a_B \approx 4$ nm, which is comparable with the average spacing between impurities at a typical impurity concentration ($\sim 10^{19}$ cm^{-3}). This may result in a considerable hopping amplitude between impurity orbitals and contribute to some conduction if this impurity band is partially occupied. Based on this model, the 2D carrier density contributed by impurities is $n_{2D} < 10^{19}$ cm^{-3} $\times$ 10 nm $= 10^{13}$ cm^{-2}. To account for the residue conduction, the mobility of such hopping is then greater than 1000 cm^{2}V^{-1}s^{-1}, which is unreasonably high. Based on a similar consideration, Ref. [3] extracted a slightly lower impurity band mobility of 380 cm^{2}V^{-1}s^{-1} but still seems too high for a hopping mechanism, which should typically be below 1 cm^{2}V^{-1}s^{-1} [4]. Moreover, hopping electrons should also contribute to Hall coefficient depending on the occupancy of the impurity band. If impurity conduction dominates in the region near the charge neutral point, tuning the surface states shouldn't cause a significant change in Hall coefficient. Although Ref.[2] did not report a Hall coefficient polarity switching, a similar experiment by [5] did report such switching in Ca-doped Bi_2Se_3 thin films. Ca-doping is expected to induce even higher impurity levels compared to exfoliated single crystals. Therefore impurity band conduction does not seem to be a good explanation of the residual conductivity.

Ref. [6] adopts another explanation which attributes the conductance residue to electron/hole puddles formed when the Fermi level is close to the Dirac point. This model inherits from a similar study in graphene which concludes that the main source of scattering in graphene is unscreened long range Coulomb scattering [7]. This long range interaction results in a surface potential fluctuation in a relatively large length scale, where electrons can be semi-classically thought to form "puddles". However, it has been demonstrated that in the most common 3D TIs such as Bi_2Se_3, the dominant impurity source is Se vacancies, which is short range and cannot be thought in terms of a semi-classical potential fluctuation. On the other hand, if the minimum conductivity $\sim 5e^2/h$ observed in [6] indeed comes from long range potential fluctuation, a brief estimation reveals that the residue carrier density is $n^* \approx 10^{12}$ cm^{-2}, which corresponds to a potential fluctuation of 120 meV. Those puddle-like residue carriers actually form a lot of mini-pn-junctions and should not be as mobile as uniform carriers. Therefore, the actually required fluctuation is even larger to account for the large σ. The potential fluctuation on the surface of Bi_2Se_3

can be directly measured through scanning tunneling spectroscopy, which has already been carried out by several groups. Ref. [8] did report a typical fluctuation of about 120 mV, but suggested this fluctuation is structural rather than disorder-induced. Moreover, the morphology does not really look like "puddles" but rather some "spikes". On the other hand, Ref. [9] reported a much smaller value around 10 mV, suggesting such potential fluctuation is quite sample-dependent and cannot be universally adopted to explain the residue conductivity.

Supplementary References

[1] Murakami, S., Nagaosa, N. & Zhang., S. C. Spin-Hall insulator. *Phys. Rev. Lett.*, **93,** 156804 (2004).

[2] Sacepe, B. *et al.* Gate-tuned normal and superconducting transport at the surface of a topological insulator. *Nature Comms.*, **2,** 575 (2011).

[3] He, L. *et al.* Surface-dominated conduction in a 6 nm thick Bi_2Se_3 thin film. *Nano Lett.*, **12,** 1486–1490 (2012).

[4] Lee, J.-S., Kovalenko, M. V., Huang, J., Chung, D. S., & Talapin, D. V. Band-like transport, high electron mobility and high photoconductivity in all-inorganic nanocrystal arrays. *Nature Nanotech.*, **6,** 348–352 (2011).

[5] Checkelsky, J. G., Hor, Y. S., Cava, R. J., & Ong., N. P. Bulk band gap and surface state conduction observed in voltage-tuned crystals of the topological insulator Bi_2Se_3. *Phys. Rev. Lett.*, **106,** 196801 (2011).

[6] Kim, D. *et al.* Surface conduction of topological dirac electrons in bulk insulating Bi_2Se_3. *Nature Phys.*, **8,** 459–463 (2012).

[7] Adam, S., Hwang, E. H., Galitski, V. M., & Das Sarma., S. A self-consistent theory for graphene transport. *Proc. Natl Acad. Sci. USA*, **104,** 18392–18397 (2007).

[8] Liu, Y. *et al.* Charging dirac states at antiphase domain boundaries in the three-dimensional topological insulator Bi_2Se_3. *Phys. Rev. Lett.*, **110,** 186804 (2013).

[9] Beidenkopf, H. *et al.* Spatial fluctuations of helical dirac fermions on the surface of topological insulators. *Nature Phys.*, **7,** 939–943 (2011).
